# Dr. George Julius Engelmann

**Published:** 1903-12

**Authors:** 


Dr. George Julius Engelmann, of Boston, died at Nashua, N. H., from pneumonia, November 16, 1903, aged 56 years. He graduated in arts at Washington University in 1867, and afterward pursued his medical studies in Europe, taking his doctorate degree from the University of Berlin in 1871, and that of Master of Obstetrics at Vienna in 1873. He practised and taught in Saint Louis, from 1873 to 1897, and then removed to Boston, where he has since resided. He was known as a gynecologist, and was very much interested in archeology and ethnology. He was president of the Southern Surgical and Gynecological Association in 1890, and of the American Gynecological Society in 1900.

